# Antibacterial peptide Reg4 ameliorates *Pseudomonas aeruginosa*-induced pulmonary inflammation and fibrosis

**DOI:** 10.1128/spectrum.03905-23

**Published:** 2024-03-19

**Authors:** Xiaoyu Wan, Weipeng Wang, Jing Zhu, Yongtao Xiao

**Affiliations:** 1 Department of Respiratory and Critical Care Medicine, Shanghai Pulmonary Hospital, School of Medicine, Tongji University, Shanghai, China; 2 Xin Hua Hospital, School of Medicine, Shanghai Jiao Tong University, Shanghai, China; The University of North Carolina at Chapel Hill, Chapel Hill, North Carolina, USA

**Keywords:** *Pseudomonas aeruginosa*, pulmonary fibrosis, Reg4, antimicrobial peptides, macrophage

## Abstract

*Pseudomonas aeruginosa* (*P. aeruginosa*) is a Gram-negative facultative anaerobe that has become an important cause of severe infections in humans, particularly in patients with cystic fibrosis. The development of efficacious methods or mendicants against *P. aeruginosa* is still needed. We previously reported that regenerating islet-derived family member 4 (Reg4) has bactericidal activity against *Salmonella* Typhimurium, a Gram-negative flagellated bacterium. We herein explore whether Reg4 has bactericidal activity against *P. aeruginosa*. In the *P. aeruginosa* PAO1-chronic infection model, Reg4 significantly inhibits the colonization of PAO1 in the lung and subsequently ameliorates pulmonary inflammation and fibrosis. Reg4 recombinant protein suppresses the growth motility and biofilm formation capability of PAO1 *in vitro*. Mechanistically, Reg4 not only exerts bactericidal action via direct binding to the *P. aeruginosa* cell wall but also enhances the phagocytosis of alveolar macrophages in the host. Taken together, our study demonstrates that Reg4 may provide protection against *P. aeruginosa*-induced pulmonary inflammation and fibrosis via its antibacterial activity.

**IMPORTANCE:**

Chronic lung infection with *Pseudomonas aeruginosa* is a leading cause of morbidity and mortality in patients with cystic fibrosis. Due to the antibiotic resistance of *Pseudomonas aeruginosa*, antimicrobial peptides appear to be a potential alternative to combat its infection. In this study, we report an antimicrobial peptide, regenerating islet-derived 4 (Reg4), that showed killing activity against clinical strains of *Pseudomonas aeruginosa* PAO1 and ameliorated PAO1-induced pulmonary inflammation and fibrosis. Experimental data also showed Reg4 directly bound to the bacterial cell membrane and enhanced the phagocytosis of host alveolar macrophages. Our presented study will be a helpful resource in searching for novel antimicrobial peptides that could have the potential to replace conventional antibiotics.

## INTRODUCTION

*Pseudomonas aeruginosa* (*P. aeruginosa*) is a Gram-negative bacterium and an important opportunistic pathogen (1). *P. aeruginosa* is commonly associated with nosocomial infections and causes various respiratory infections (2–6), including healthcare-associated pneumonia, ventilator-associated pneumonia, and cystic fibrosis. The increasing risk of antimicrobial resistance against *P. aeruginosa*, including multi-drug resistance (MDR), has dramatically impaired the treatment strategies (7–9). Naturally occurring antimicrobial peptides have demonstrated promising results in treating MDR bacteria (10–13). In this respect, antimicrobial peptides appear to be an interesting alternative to combat *P. aeruginosa*.

Regenerating islet-derived 4 (Reg4) is a member of the Reg family, which was discovered and characterized in a cDNA library of ulcerative colitis (14). The human REG4 gene is located in Chromosome 1 (1q12-q21) and contains an open reading frame of 474 bp. The REG4 protein contains 158 amino acids with a predicted molecular mass of 18.2 kDa. It shares 39% protein sequence identity with Reg3, 38% with Reg1α, and 39% with Reg 1β (14). The N-terminal signal peptide of REG4 is predicted to have about 22 amino acids and it contains a cleavage site between VLG and D (14). REG4 has pro-proliferative, differentiation-inducing, and antiapoptotic potential (15) and is probably involved in lectin-related biological processes by containing a sequence motif homologous to the calcium-dependent (C-type) lectin-like domain (15, 16). We previously reported that Reg4 acts as a novel antimicrobial peptide and performs bactericidal action against *Salmonella* via a motif (HDPQK) homologous to a calcium-dependent (C-type) lectin-like domain (17). Moreover, Qi et al. (18) have showed that Reg4 stimulated complement-mediated attack complexes to prevent the overgrowth of *Escherichia coli* in the mouse gut. However, very little is known regarding the role of Reg4 in *P. aeruginosa* infections. In this study, we aimed to investigate the exact roles of Reg4 *in vivo* and *ex vivo* and the underlying mechanisms during *P. aeruginosa* infection of the lungs.

## RESULTS

### Reg4 increases in response to *P. aeruginosa* infection

**Fig 1:**
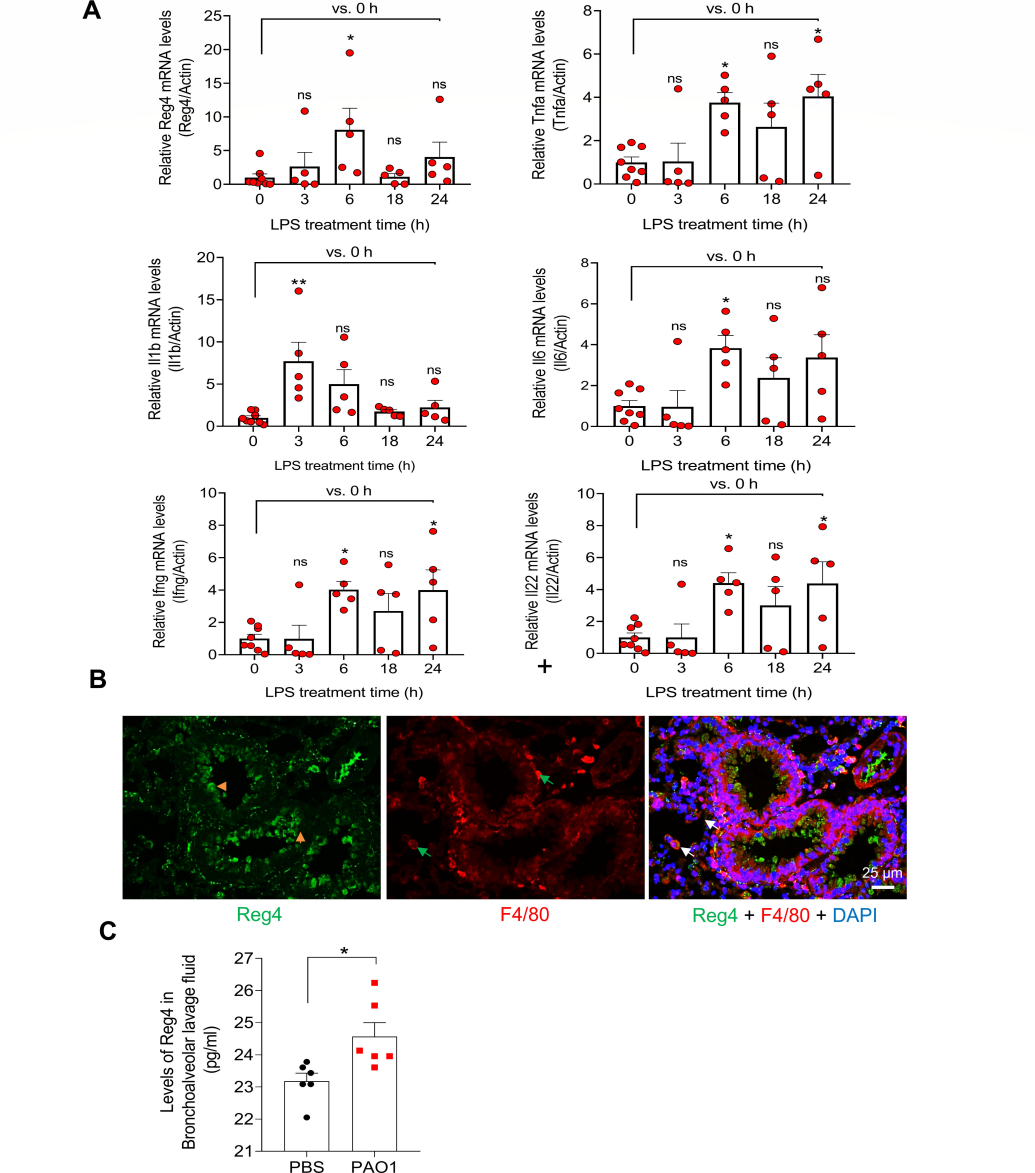
Reg4 expression by LPS or PAO1 stimulation (**A**) Quantification of Reg4 mRNA and inflammatory markers in the lungs in response to LPS challenge at the indicated time points *n* = 5–8 mice per group. (**B**) Immunofluorescence staining for Reg4 and F4/80 in the mice lung, *n* = 4. White arrows indicate costaining of Reg4 and F4/80. Yellow arrows indicate Reg4-positive ciliated cells. Green arrows show F4/80-positive cells. DAPI staining indicates blue. (**C**) Enzyme-linked immunosorbent assay for Reg4 levels in bronchoalveolar lavage fluid of mice after 28 days of PAO1 infection. *n* = 6 mice per group. Data are mean ± standard error of the mean ; Student’s *t*-test for panel C and two-way analysis of variance for panel A were used for statistical analysis; ns, not significant (*P* ≥ 0.05); *P < 0.05 and **P < 0.01.

Purified bacterial lipopolysaccharide (LPS) was used to induce lung inflammation in mice. In the LPS-induced lung inflammation model of mice, *Reg4* mRNA level peaked at 6 hours and then gradually decreased in the lung after LPS treatment (Fig. 1A). Proinflammatory genes, including interferon-gamma (Ifng), tumor necrosis factor-alpha (Tnfa), interleukin-1 beta (Il-1b), interleukin-6 (Il-6), and interleukin-22 (Il-22), had similar expression patterns to those of Reg4 mRNA (Fig. 1A). Immunofluorescence (IF) staining showed that Reg4 was mainly expressed in the ciliated cells and colocalized with the macrophage marker F4/80 (Fig. 1B). Furthermore, Reg4 protein levels in bronchoalveolar lavage fluid (BALF) increased slightly in a chronic model of mice infected with *P. aeruginosa* PAO1 (Fig. 1C).

### Reg4 protects mice from *P. aeruginosa*-induced pulmonary inflammation and fibrosis

**Fig 2:**
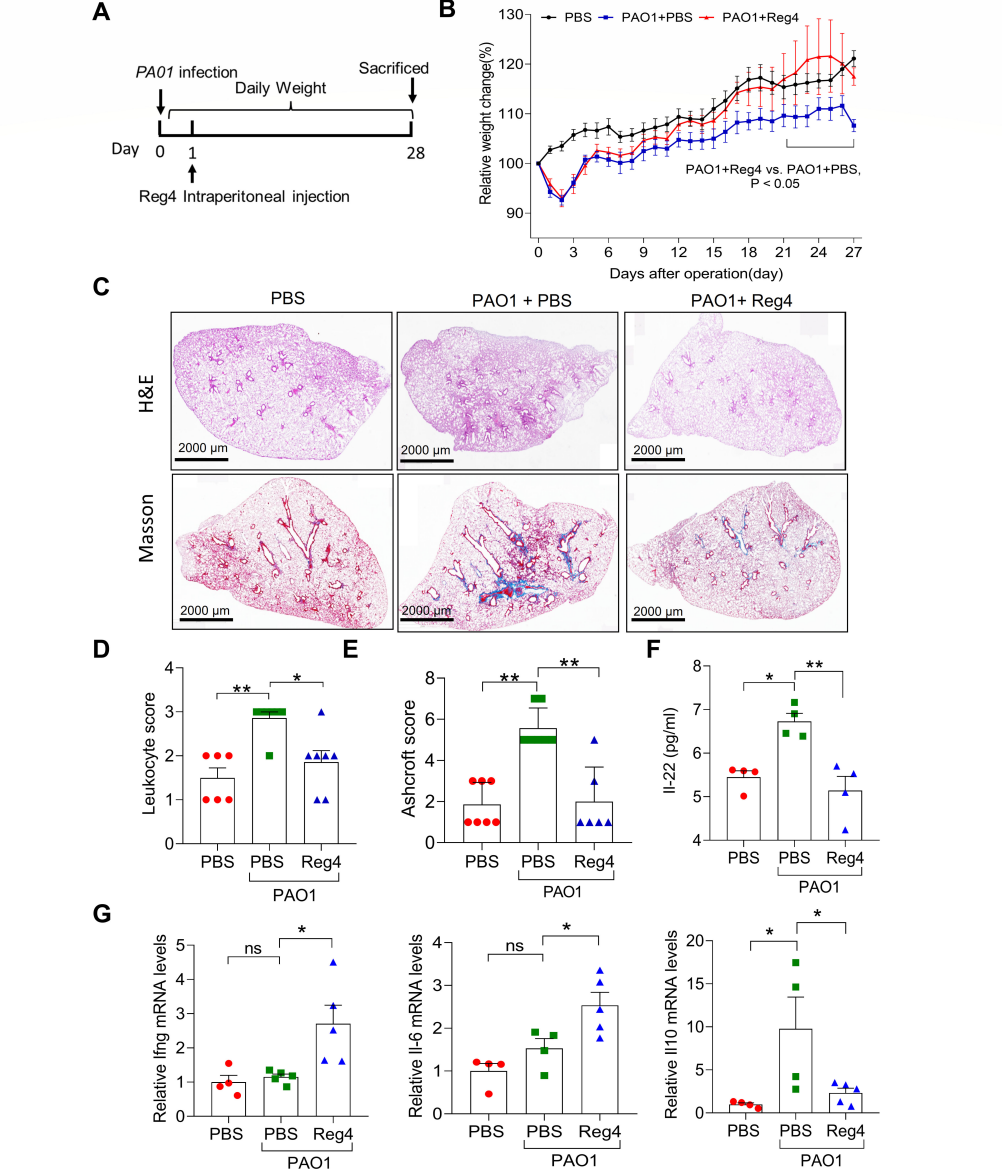
The effect of Reg4 in a chronic PAO1-induced lung infection model of mice. (**A**) Schematic illustration of the experimental procedures for mice treated with Reg4. (**B**) Comparison of the body weight loss in phosphate-buffered saline-treated, PAO1*-*infected, and PAO1*-*infected with Reg4 injected (50 µg/kg of body weight, daily) mice. (*n* = 8 per group). (**C**) Representative images of hematoxylin–eosin (H&E) and Masson’s trichrome-stained lungs in different groups. (**D**) Leukocyte infiltration score in lungs from mice in different groups (*n* = 6–7 per group). (**E**) The quantification of lung fibrosis is shown as an Ashcroft score (*n* = 6–7 per group). (**F**) ELISA for serum Il-22 levels of mice (*n* = 4 per group). (**G**) The mRNA levels of Ifng, Il-6, and Il-10 using quantitative real-time polymerase chain reaction. Values were normalized with respect to Actb expression. Data are mean ± SEM; one-way and two-way analysis of variance were used for statistical analysis in panels B and D–G, respectively; ns, not significant (*P* ≥ 0.05); *P < 0.05; **P < 0.01, *** *P* < 0.001; and ****P < 0.0001.

To further investigate the possible protective roles of Reg4 in pulmonary inflammation and fibrosis induced by *P. aeruginosa*, a PAO1-infected mouse model was established as previously reported (19). After 1 day of PAO1 infection, mice were injected with recombinant Reg4 protein daily at a dose of 50 µg/kg of body weight (Fig. 2A). As shown in Fig. 2B through D, PAO1 infection decreased body weight and induced lung lesions in phosphate-buffered saline (PBS) control mice but not in the Reg4-treated mice (Fig. 2B through D). The histological scores of the lungs were improved when the infected mice were dosed with Reg4 (Fig. 2D). Furthermore, Reg4 treatment inhibited pulmonary fibrosis, which is characterized by decreasing Ashcroft scores in PAO1-infected mice (Fig. 2E). An enzyme-linked immunosorbent assay (ELISA) assay revealed a higher level of serum Il-22 in PAO1-infected mice than in uninfected mice but not in PAO1-infected mice treated with Reg4 protein (Fig. 2F). Compared to PAO1-infected mice, mice treated with Reg4 showed lower expression of interleukin-10 (Il-10) and higher expression of Ifng, Il-6, C-X-C motif chemokine ligand 2 (Cxcl2), NF-kappaB inhibitor alpha (Nfkbia), and TNF-alpha-induced protein 3 (Tnfaip3) (Fig. 2G; Fig. S1).

### Reg4 reduces colonization and translocation of *P. aeruginosa*

**Fig 3:**
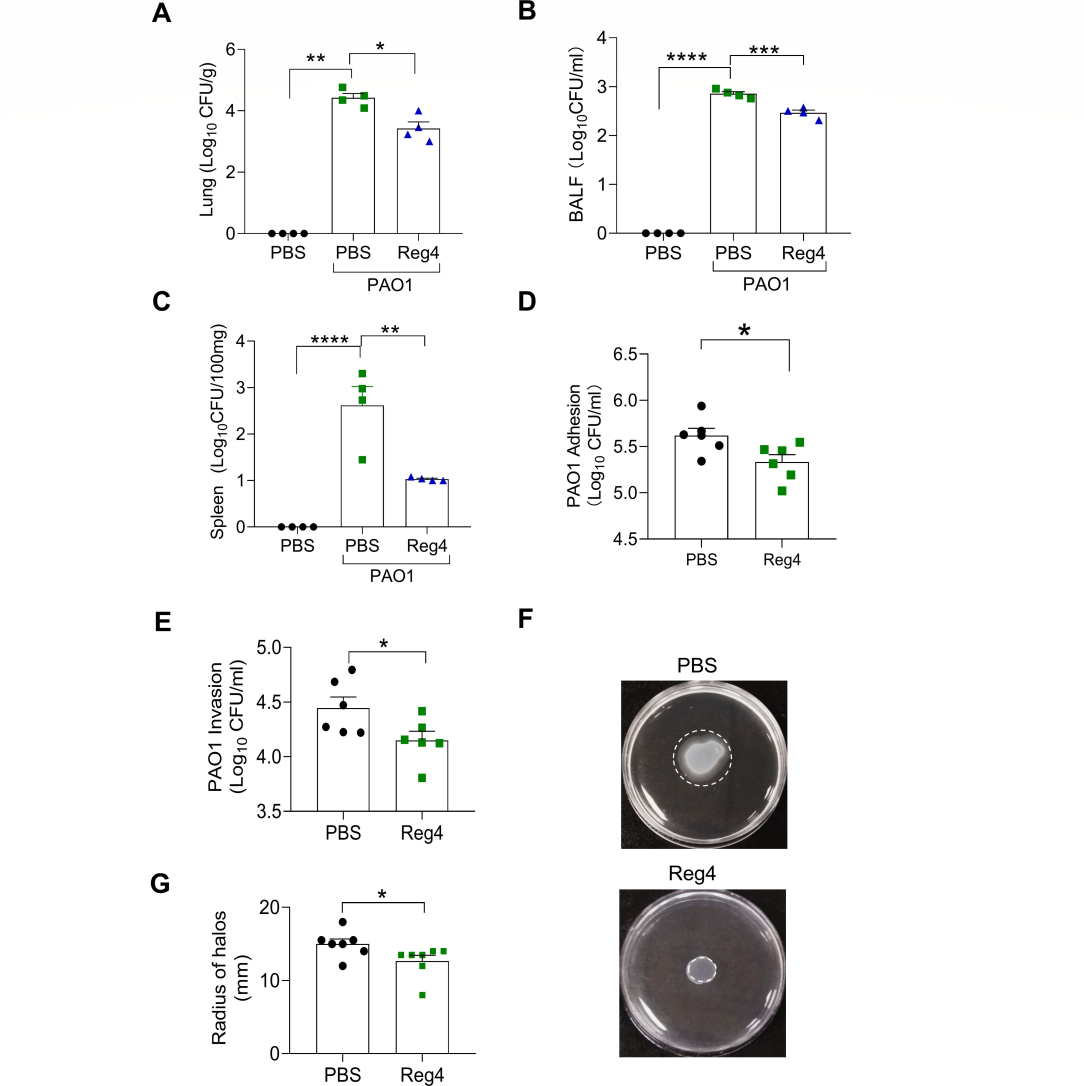
Reg4 reduces the adhesion and invasion of PAO1 on lung epithelial cells (**A**) Quantification of CFUs in mice lungs (*n* = 4 per group). (**B**) Quantification of CFUs in mice BALF (*n* = 4 per group). (**C**) Quantification of CFUs in the spleen (*n* = 4 per group). (**D**) Quantification of CFUs for the adhesion of PAO1 to murine lung epithelial (MLE-12) cells treated with PBS or Reg4 (*n* = 4 per group). (**E**) Quantification of CFUs for the invasion of PAO1 to MLE-12 cells treated with PBS or Reg4 (*n* = 4 per group). (**F**) Representative images of the swarming activity of PAO1 incubated with PBS or Reg4 after 10 hours. (**G**) Quantification of the length of circular swarms in panel F (*n* = 7 per group). Data are mean ± SEM; analysis of variance was used for panels A–C; Student’s *t*-test for panels D, E, and G; ns, not significant (*P* ≥ 0.05); *P < 0.05; **P < 0.01; ***P < 0.001; and ****P < 0.0001.

Next, we investigated the role of Reg4 in PAO1 colonization in the lungs. We first detected the number of viable PAO1 cells in the lung and BALF samples from phosphate-buffered saline, PBS + PAO1, and Reg4 + PAO1 mice. Compared to PBS + PAO1 mice, Reg4 + PAO1 mice had a smaller number of PAO1 in the lungs and BALF (Fig. 3A and B; Fig. S2A). To further investigate the effects of Reg4 on PAO1 translocation to the spleen, the number of colony-forming units (CFUs) in the spleen was determined. Reg4 + PAO1 mice had significantly decreased numbers of viable PAO1 in the spleen compared to that of PBS + PAO1 mice (Fig. 3C; Fig. S2A).

We further tested whether Reg4 could inhibit the adhesion and invasion of PAO1 in murine lung epithelial (MLE-12) cells. MLE-12 cells were incubated with PAO1 with or without Reg4, and PAO1*-*attached or -invaded cells were detected. As shown in Fig. 3D and Fig. S2B, the mean number of CFU of PAO1 attached to MLE-12 cells was significantly decreased by Reg4 treatment. Additionally, after killing the bacteria adhered to the cell surface with gentamicin, less intracellular PAO1 was found in the Reg4-treated group than in the PBS-treated group (Fig. 3E; Fig. S2C). Flagella-mediated surface movements, such as swimming and swarming, play a determining role in initial bacterial attachment and colonization processes (20). Here, we investigated whether Reg4 affects PAO1 infection by affecting bacterial motility. PAO1 was cultured with Reg4, added to the center of semi-solid agar, and incubated for 10 hours. As expected, the migratory activity of PAO1 was inhibited by Reg4 (Fig. 3F and G).

### Reg4 has bactericidal activity against *P. aeruginosa*

**Fig 4:**
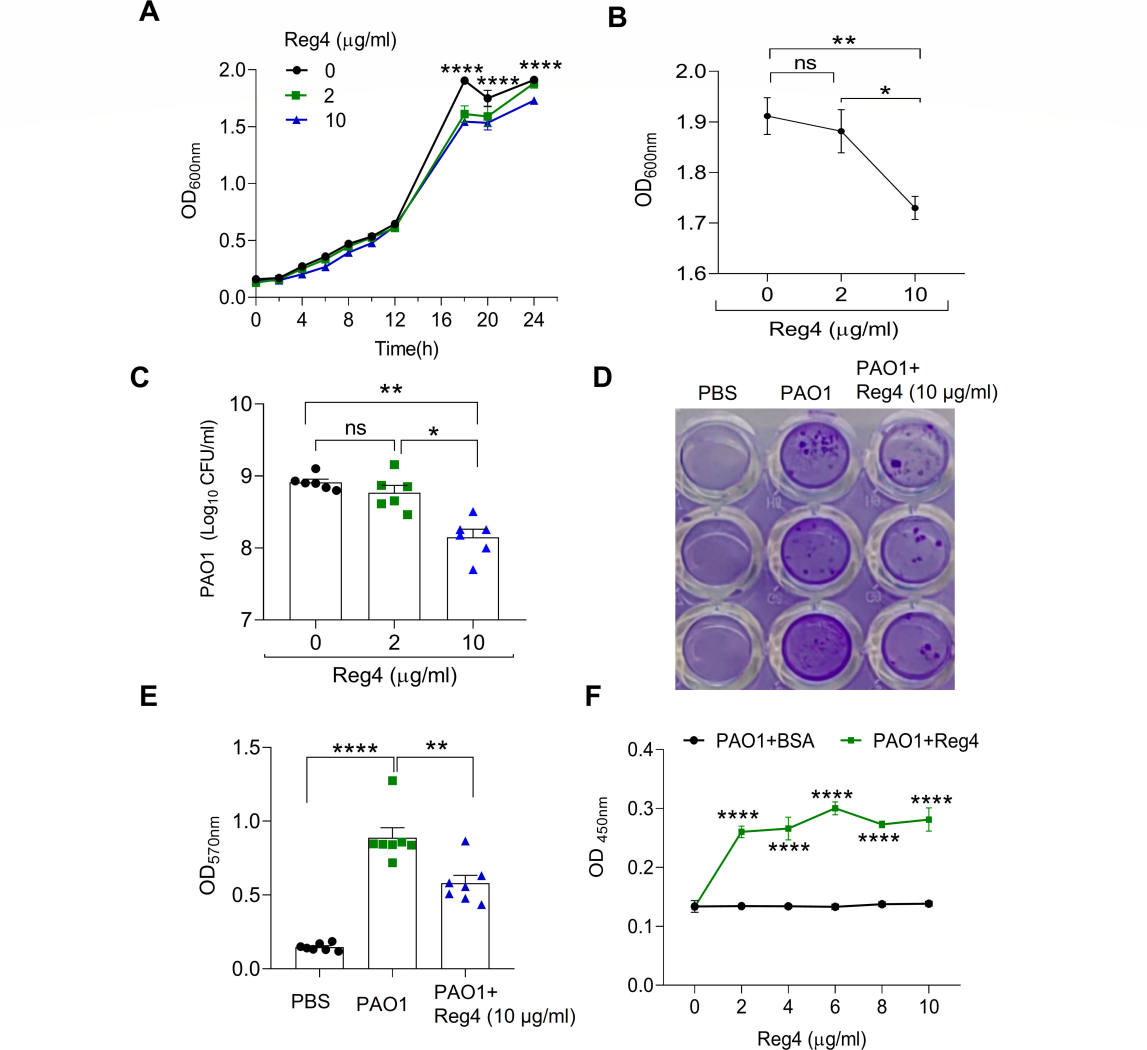
Antimicrobial activity of Reg4 against PAO1. (**A**) Growth curve of PAO1 treated with the indicated concentrations of Reg4 (0, 2, and 10 µg/mL) at the indicated time points. Bacterial growth was determined by measuring absorbance at 600 nm (*n* = 7–8 per group). (**B**) Growth densities of PAO1 treated with varying concentrations of Reg4 (0, 2, and 10 µg/mL) at 24 hours (*n* = 7–8 per group). (**C**) Quantification of CFUs for PAO1 treated with the indicated concentrations of Reg4 (0, 2, and 10 µg/mL; *n* = 6). (**D**) Representative image of biofilm formation of PAO1 treated with PBS or Reg4 (10 µg/mL). (**E**) Quantification of biofilm in panel D (*n* = 8). (**F**) ELISA of Reg4 binding with PAO1. Data are mean ± SEM; analysis of variance was used for panels A, B, C, E, and F; ns, not significant; * *P* < 0.05; **P < 0.01, ***P < 0.001; and ****P < 0.0001.

We purified recombinant mouse Reg4 and incubated it with PAO1 to determine its effect on the growth of PAO1. As shown in Fig. 4A and B, the growth of PAO1 was inhibited by Reg4 in a dose-dependent manner. Reg4 dose-dependently decreased the number of PAO1 after 24 hours (Fig. 4C). PAO1 can form biofilms that protect them from physical eradication and chemical elimination (21). Reg4 treatment significantly inhibited biofilm formation by PAO1 (Fig. 4D and E). Furthermore, ELISA showed that Reg4, but not bovine serum albumin, could bind with PAO1 in a dose-dependent manner (Fig. 4F).

### Reg4 alters polarization and phagocytosis of alveolar macrophages

**Fig 5:**
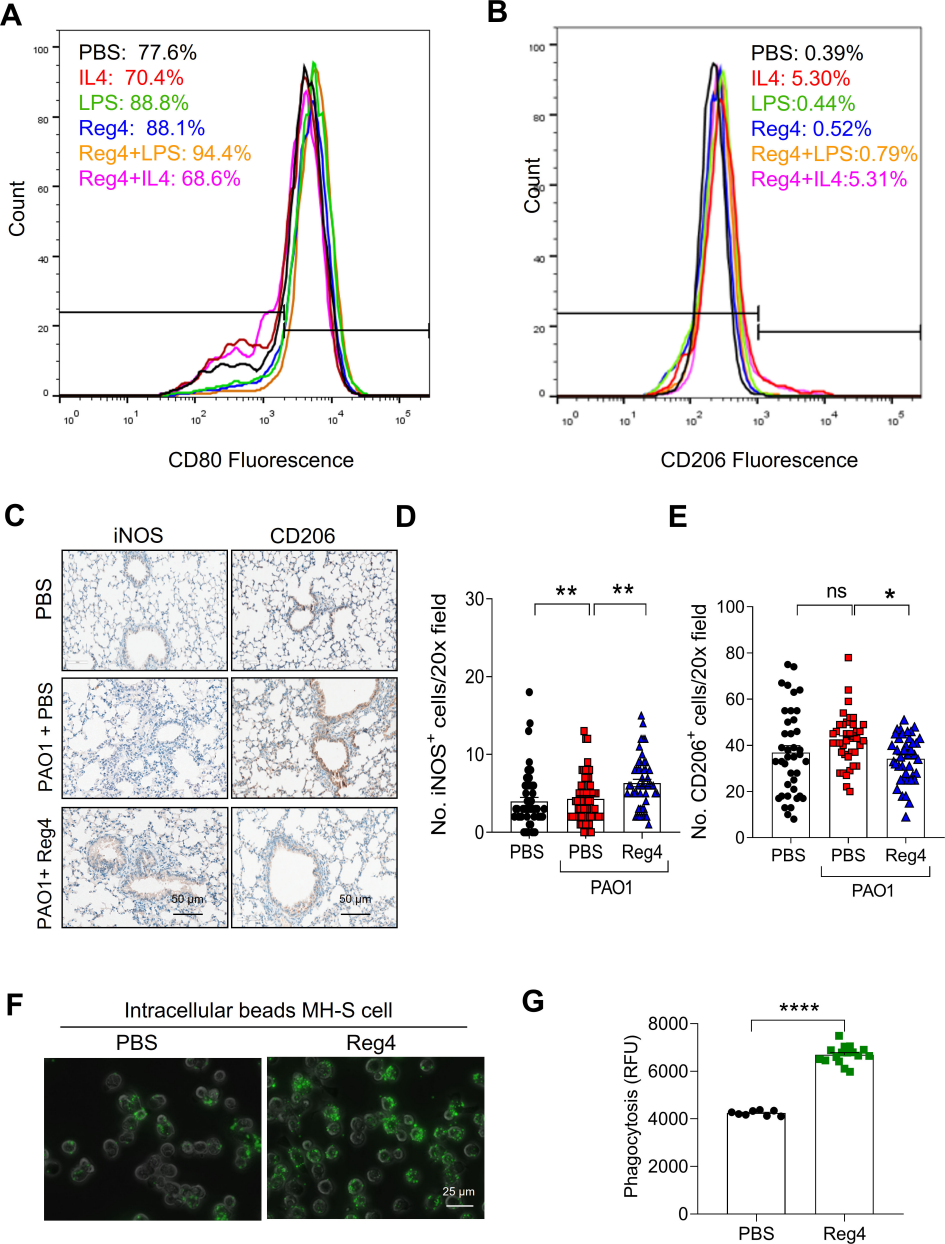
Reg4 promotes polarization and phagocytosis of alveolar macrophages. Representative fluorescence-activated cell sorting plot to detect CD80 (**A**) and CD206 (**B**) in macrophages treated with Reg4, LPS, or IL-4. (**C**) Representative images of IHC staining of iNOS and CD206 in mice lung tissues (*n* = 4 per group). (**D and E**) Quantification of iNOS and CD206 in mice lung tissues in panel C (20× microscope field). (**F**) Representative images of phagocytic uptake of latex beads by PBS and Reg4-treated macrophages (MH-S cells). (**G**) Quantification of panel F. Data are mean ± SEM; analysis of variance was used for panels D–E; Student’s *t*-test for panel G; ns, not significant; *P < 0.05; ***P < 0.01; and ****P < 0.0001.

Activated macrophage infiltration plays an important role in the occurrence and development of lung injury. M1 and M2 are the major subtypes of macrophages. In this study, CD80 and CD206 were detected in an alveolar macrophage cell line (MH-S) to examine the state of macrophage polarization. The number of M1 macrophages increased upon LPS stimulation and decreased upon interleukin-4 (Il-4) stimulation (Fig. 5A). Reg4 treatment increased the number of CD80-labeled M1 macrophages (Fig. 5A). Moreover, Reg4 treatment increased the number of CD80-labeled M1 macrophages (Fig. 5A). In contrast, Reg4 treatment decreased the number of CD206-labeled M2 macrophages (Fig. 5B). Consistently, the immunohistochemical (IHC) staining showed that Reg4 treatment increased the recruitment of iNOS-labeled M1 macrophages and reduced the recruitment of CD206-labeled M2 macrophages (Fig. 5C through E). Subsequently, the phagocytic capacity of macrophages was evaluated by measuring the phagocytosis of carboxylate-modified polystyrene latex beads. As shown in Fig. 5F and G, Reg4 treatment enhanced the phagocytosis of MH-S with more intracellular beads and a higher intracellular fluorescence intensity.

### Reg4 impairs extracellular acidification rate in alveolar macrophages

**Fig 6:**
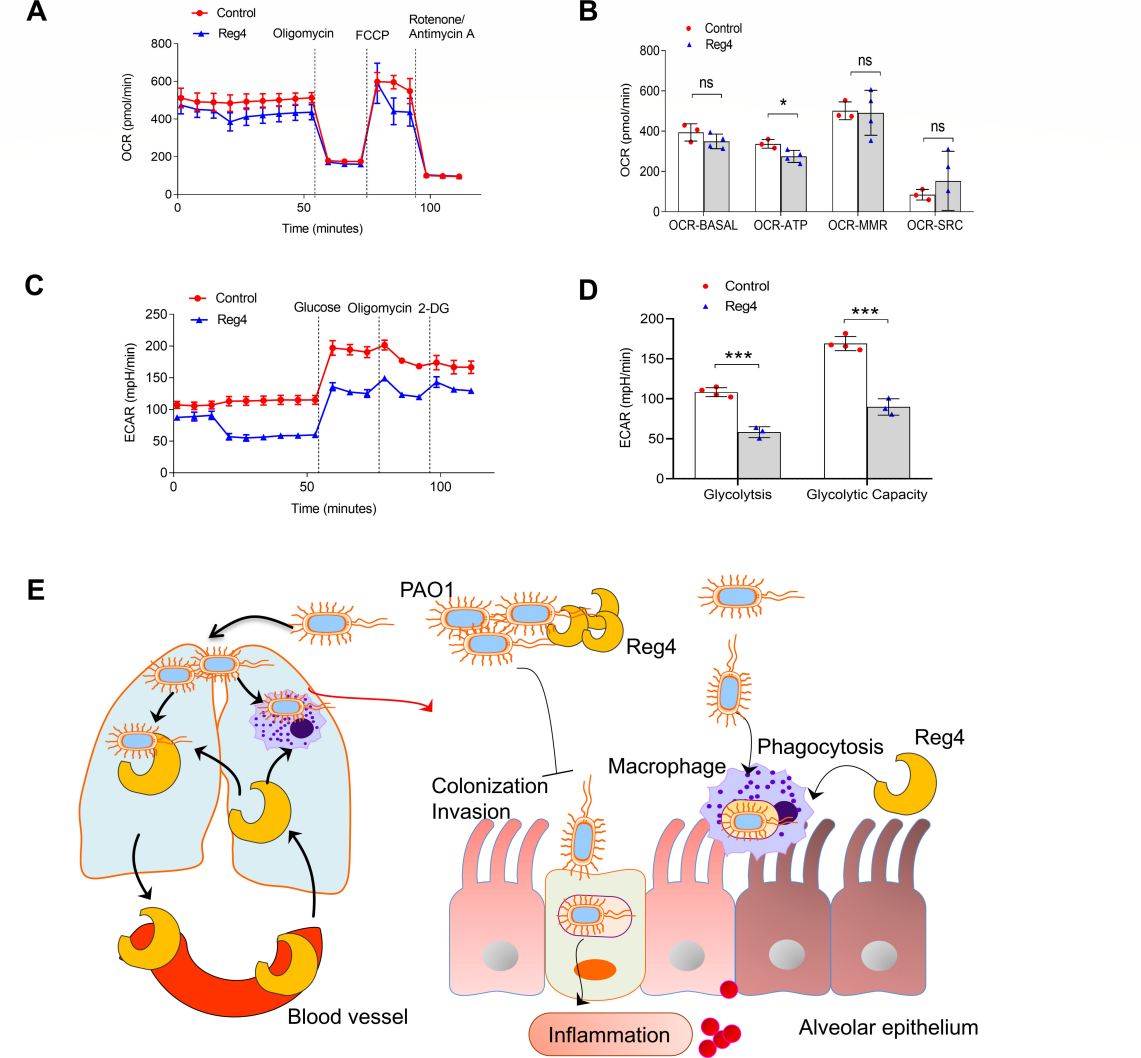
Reg4 reduced ECAR in alveolar macrophages. (**A**) Representative OCR experiments were analyzed in mouse alveolar macrophage MH-S treated with PBS or Reg4 using the Seahorse XF96 extracellular flux analyzer. (**B**) Levels of mitochondrial respiratory function: basal respiration (OCR-BASAL), ATP production (OCR-ATP), maximal respiration (OCR-MMR), and spare respiratory capacity (OCR-SRC) in MH-S cells. (**C**) Representative ECAR experiments performed on MH-S cells treated with PBS or Reg4. (**D**) Levels of glycolysis and maximal glycolytic capacity in MH-S cells. (**E**) Schematic diagram of potential mechanisms of Reg4-mediated bactericidal effects on PA infection. Data are mean ± standard error of the mean. Student’s *t*-test was used for panels B and D; ns, not significant; *P < 0.05; and ***P < 0.001.

Using a Seahorse XF96 Analyzer, we first investigated the effects of Reg4 on the oxygen consumption rate (OCR) in alveolar macrophages. OCR is an indicator of mitochondrial respiratory capacity and energy production. Inhibitors, including Oligomycin A, FCCP, and rotenone/antimycin A, were used to measure oxygen consumption during different mitochondrial processes. Oxygen consumption linked to mitochondrial ATP production (OCR-ATP) can be determined by adding the ATP synthase inhibitor oligomycin A. OCR-ATP production in MH-S cells was suppressed by Reg4 (Fig. 6A and B). We further measured the extracellular acidification rate (ECAR) of MH-S cells. Glucose (5  mM) was added to increase the ECAR, which is the rate of glycolysis under basal conditions (Fig. 6C and D ). A significantly decreased rate of glycolysis was observed in Reg4-treated MH-S cells compared to that of the PBS control (Fig. 6C and D). The subsequent addition of oligomycin A shifts the energy production to glycolysis and causes a further increase in the ECAR, which uses maximum glycolytic capacity. We observed a significantly reduced maximum glycolytic capacity in Reg4-treated MH-S cells compared to that of the control (Fig. 6C and D).

## DISCUSSION

The severity of the health threat posed by antibiotic-resistant *P. aeruginosa* has prompted the development of new antimicrobial therapies for *P. aeruginosa*. Here, we identified Reg4 as a new peptide that exerts antimicrobial activity against *P. aeruginosa* both *in vitro* and *in vivo*. Additionally, Reg4 may mediate the polarization of M1 macrophages to enhance phagocytosis and clearance of PAO1 (Fig. 6E).

The ability of *P. aeruginosa* to form biofilms is a critical factor that causes severe and recalcitrant infections that are associated with significant morbidity and mortality (22). Biofilms provide *P. aeruginosa* an enormous advantage by promoting survival on artificial materials, evasion from the immune system, and tolerance to antimicrobial therapy (23). *P. aeruginosa* exhibits a characteristic flagellum-driven locomotory social trait called swarming, quorum-dependent motility that relies on Rhl and Las quorum-sensing systems (24). In this study, we demonstrated that Reg4 could bind to *P. aeruginosa* and inhibit its swarming activity, thereby effectively restraining biofilm formation by *P. aeruginosa*. The epithelial barrier provides the first line of defense against *P. aeruginosa-*induced lung infection. Patients with damaged or disrupted epithelium, such as intubated patients, are at an increased risk of developing *P. aeruginosa* infections. The epithelial barrier provides a physical barrier to bacterial invasion through a network of cell–cell contacts, including tight junctions. In addition, mucociliary clearance in the upper respiratory tract prevents the establishment of *P. aeruginosa* infection (25). Here, we found that Reg4 administration increased the clearance of *P. aeruginosa* from the lungs and spleen. As a result, Reg4 treatment resulted in a less severe inflammatory reaction caused by *P. aeruginosa*. These findings suggest that Reg4 exerts bactericidal activity against *P. aeruginosa* by reducing biofilm formation and invading the epithelial barrier of the lung. Antimicrobial peptides are characterized by broad-spectrum antimicrobial activity, killing potential, high selectivity, and low toxicity and are found to be involved in a variety of biological functions, including immune regulation and wound healing (26). We found that Reg4 exerted bactericidal activity against PAO1 at a dose of 10 µg/mL *in vitro* and 50 µg/kg of body weight *in vivo*, which is consistent with our previous study (17). However, further study is needed to determine the minimum bactericidal concentration of Reg4.

Significant evidence has demonstrated the importance of innate immune cells in the host response to infections induced by the biofilm of *P. aeruginosa* (27). Alveolar macrophages, which are important mediators of the innate immune response to *P. aeruginosa*-induced lung infections, are responsible for the phagocytosis of bacteria and the recruitment of additional immune cells. Macrophages with different polarization states show diverse immunological responses. Detailed analysis of the different macrophage subpopulations revealed that Reg4 induced proinflammatory M1-like macrophage polarization, suggesting that Reg4 promoted the classical activation of macrophages. M1 macrophages are traditionally regarded as phagocytes with clear debris and bacterial pathogens (28). Reg4 stimulated phagocytosis and killing of *P. aeruginosa*. Recent evidence has shown the crucial role of metabolic reprogramming in regulating macrophage polarization (29, 30). LPS-induced classically activated M1 macrophages have been observed by a reduced ECAR (29). Consistently, we found that Reg4 significantly decreased glycolysis in the macrophages. We propose that Reg4-induced M1 macrophage polarization may be mediated by a reduction in the ECAR.

In conclusion, this is the first study to notice that Reg4 exerts antimicrobial activity against *P. aeruginosa in vitro* and *in vivo*. Reg4 can inhibit the motility and biofilm formation of *P. aeruginosa*, reducing its colonization in the lungs. Furthermore, Reg4 can stimulate macrophages toward M1 polarization to defend against invading *P. aeruginosa*. Therefore, Reg4 may be a promising therapeutic agent for treating *P. aeruginosa*-associated pneumonia.

## MATERIALS AND METHODS

### Animals

Wild-type male C57BL/6 mice were purchased from Shanghai Jihui Laboratory Animal Care Co., Ltd. (Shanghai, China) and housed under specific pathogen-free conditions at the Department of Model Animal Research, Xinhua Hospital Affiliated to the Shanghai Jiao Tong University School of Medicine.

### Chronic lung infection mouse models

Eight-week-old male C57BL/6 mice (*n* = 12–18 per group) were randomly divided into three groups as follows: PBS group, PAO1 group (infected with PAO1), and PAO1 + Reg4 group (infected with PAO1 and treated with Reg4 at a dose of 50 µg/kg of body weight). Infection experiments were performed as previously described (31). Briefly, agar-bead-embedded bacterial cells were prepared and enumerated. Then, the agar beads were diluted into the same inoculum of 4.0 × 10^6^ CFU in 50 µL of sterile saline and then intranasally instilled into anesthetized C57BL/6 mice. The weight change of mice was recorded by two observers blinded to the groups. Mice were treated with PBS or Reg4 (50 µg/kg of body weight) daily by intraperitoneal injection from the first day after infection. Mice were euthanized on day 28.

### Histopathology and lung inflammation scoring

The whole lungs were aseptically removed from each mouse and fixed in 4% formaldehyde and stained with hematoxylin and eosin staining. The leukocyte score was determined by two blinded investigators according to the previous report (32, 33). Briefly, 1 indicates mild, scattered leukocytes in the interstitium and airspaces; 2 indicates moderate, scattered leukocytes forming aggregates in the interstitium and perivascular spaces and extending into the airspaces; and 3 indicates moderate to severe prominent leukocyte infiltration in the interstitium, extending into the airspaces and often with prominent perivascular aggregates (32, 33). For collagen staining, the lungs were stained with Masson’s trichrome. The lungs were scored to estimate the severity of pulmonary fibrosis according to the Ashcroft score method (34).

### Assays for Reg4 bactericidal effects against *P. aeruginosa*

The bactericidal effects of Reg4 were determined using the procedures from both the Clinical and Laboratory Standards Institute and the European Committee on Antimicrobial Susceptibility Testing (35–37). The detailed procedures are listed in the supplemental material.

### Statistical analysis

Data are presented as mean ± standard error of the mean and were analyzed using GraphPad Prism v.8 (GraphPad, San Diego, CA, USA). Two groups were compared using two-tailed unpaired Student’s *t*-test (parametric) or Mann–Whitney *U* test (nonparametric). Multiple groups were compared using one-way analysis of variance (ANOVA) with Tukey’s test (parametric) or Kruskal–Wallis with Dunn’s test (nonparametric). Differences in loss of body weight were analyzed using two-way repeated-measures ANOVA. A *P*-value < 0.05 was considered statistically significant. The detailed Materials and Methods are given in supplemental material.

## SUPPLEMENTAL MATERIAL

Supplemental material spectrum.03905-23-s0001.docx Supplemental methods, Tables S1 and S2, and Figures S1 and S2.

ASM does not own the copyrights to Supplemental Material that may be linked to, or accessed through, an article. The authors have granted ASM a non-exclusive, world-wide license to publish the Supplemental Material files. Please contact the corresponding author directly for reuse.
