# Supplementary material for: Antibacterial peptide Reg4 ameliorates Pseudomonas aeruginosa-induced pulmonary inflammation and fibrosis
Source: Microbiol Spectr. 2024 Mar 19;12(5):e03905-23. doi: 10.1128/spectrum.03905-23 (PMC11064540; doi:10.1128/spectrum.03905-23)
Supplement: Supplemental material — Supplemental methods, Tables S1 and S2, and Figures S1 and S2. [file spectrum.03905-23-s0001.docx]

**Antibacterial peptide Reg4 ameliorates *Pseudomonas aeruginosa*-induced pulmonary inflammation and fibrosis**

Xiaoyu Wan, Weipeng Wang,Jing Zhu, Yongtao Xiao

**Table of content**

Supplemental Materials and Methods, Page 2 - 7

Supplemental Tables 1 – 2, Page 8 - 10

Supplemental Figures 1 – 2, Page 11 - 12

Supplementary References, Page 13 - 14

**Supplemental Materials and Methods**

**Bacteria**

The bacterial strain and materials were listed in Key Resource Table (Supplemental Table 1). PAO1 were obtained from Beijing Biobw Biological Technology Co. Ltd. (Beijing, China). PAO1 were cultured overnight in Lysogeny broth (LB) (5 g/l NaCl, 5 g/l yeast extract, and 10 g/l tryptone). The colonies after incubation were counted and expressed as colony forming units per mL of culture (CFU/ ml).

**Production of recombinant Reg4 protein**

The recombinant Reg4 protein was purified as described previously (1, 2). Briefly, the coding sequence of DNA, mouse Reg4 (NM_026328-6His), was cloned into the pET-28 Expression vector (Shanghai Genechem Co., Ltd) with an N-terminal 6-His tag and then transformed into BL21(DE3) competent cells. The expression of Reg4 protein was induced by adding 1 mM isopropyl-β-thiogalactopyranoside. The expressed protein from the cell culture was first purified by Nickel-nitrilotriacetic acid (Ni-NTA) Fast Start Kit according to the manufacturer’s instruction. Second, protein was immediately desalted using a PD MiniTrap G-25 column (GE Healthcare). Finally, the proteins were concentrated to about 1 mg/ml using a Vivaspin 20 centrifuge concentrator (GE HealthCare) and stored at -80 °C. The quality of the purified protein was analyzed by SDS-PAGE and analyzed by Coomassie Brilliant Blue staining.

**Bacterial growth inhibition assay *in vitro***

The effect of Reg4 on the growth of PAO1 was determined following procedures from both the Clinical and Laboratory Standards Institute (CLSI) and the European Committee on Antimicrobial Susceptibility Testing (EUCAST) (3-5). Briefly, PAO1 were cultured in LB medium at 37 °C overnight. Then, 10 μl of the bacterial culture was inoculated to a total of 200 μl of LB medium containing varying concentrations (0, 2, or 10 μg/ml) of Reg4 in 96-well plates. The growth of PAO1 was detected by OD_600_ measurement every 2 h using the microplate reader. Additionally, PAO1 (1×10^5^ CFU/ml) were incubated in 2 ml LB medium with varying concentrations (0, 2, or 10 μg/ml) of Reg4 at 37 °C. After 24 h, bacterial growth was determined by CFU counts.

**Biofilm formation assays**

The biofilm formation of PAO1 was investigated in the 96-well plates as described previously (6). Briefly, PAO1 was cultured overnight in LB medium overnight. 100 µl bacterial cultures (1.0×10^6^ CFU/ml) with or without Reg4 (10 μg/ml) were inoculated for 24 h at 37 ℃ without agitation. After cultivation, planktonic cells were removed gently, and the well was washed with sterile PBS. Biofilms were stained with 100 µl of 0.1% crystal violet for 15 min at room temperature. Biofilm-bound crystal violet was dissolved in 200 µl of 30% acetic acid and absorbance was measured at 570 nm.

**Enzyme-Linked Immunosorbent Assay (ELISA) assay of Reg4 binding to** PAO1

The binding capacity of Reg4 protein to PAO1 was determined following the previous study (7, 8). Briefly, 96-well plates were coated with PAO1 (10^8^ CFU/well) or 1% BSA overnight at 4 °C. Plates were then washed with PBS and blocked with 1% BSA in PBS for 2 h at 37 °C. Reg4 at varying concentrations from 0 - 10 μg/ml was added and incubated for 2 h at 37°C. The anti-Reg4 antibody was added and incubated for 2 h (dilution 1:1000), followed by incubation with a secondary HRP-conjugated goat anti-mouse IgG (dilution 1:5000) for 2 h. Thereafter, plates were added with tetramethylbenzidine substrate and read at 450 nm with the spectrometer.

**Intratracheal lipopolysaccharide (LPS) to mice**

The model of intratracheal LPS was performed according to the methods described in a previous study (9). Briefly, eight-week-old C57BL/6 were anesthetized with a mixture of ketamine (80mg/kg) and xylazine (20 mg/kg), lung injury was induced by intratracheal instillation of LPS (Escherichia coli 026:B6; Sigma Chemical, St. Louis, MO) at a dose of (3 μg/g) body weight. Mice were sacrificed at 3, 6, 18 or 24 hours after LPS. One mouse was sacrificed per time point to determine mRNA levels as a function of time. The lung, plasma and bronchoalveolar lavage fluid (BALF) were collected for further analysis.

**ELISA assay for Il-22 and Reg4**

The expression levels of Il-22 and Reg4 were quantified in bronchoalveolar lavage fluid (BALF) by using ELISA Kits according to the manufacturer’s instructions.

**Colonization and translocation of** PAO1 **in mice**

Lung tissue and spleen were collected and suspended in 1ml sterile PBS using a Tissue grinding device, serially diluted, and plated on LB agar plates containing kanamycin to determine the amount of viable PAO1 in organs. Bronchoalveolar lavage fluid (BALF) was obtained by washing with 600 μl of Hanks balanced salt solution in portions of 200 μl via a tracheal incision and the amount of PAO1 was determined.

**Cell culture**

Murine lung epithelial (MLE-12) cells were cultured in Dulbecco’s Modified Eagle’s Medium supplemented with 1 % streptomycin/penicillin solution and 10 % Fetal Bovine Serum. MH-S, a murine alveolar macrophage cell line, cultured in Roswell Park Memorial Institute (RPMI)-1640 medium with 1 % streptomycin/penicillin solution and 10 % Fetal Bovine Serum. The cells were maintained routinely in a humidified incubator of 95% air and 5% carbon dioxide at 37 °C.

**Bacterial adhesion and invasion assay on MLE-12 cells**

The bacterial adhesion and invasion assay on MLE-12 cells was performed as previously described (7, 10). Briefly, bacterial strains were prepared and added to MLE-12 cells (2.5 × 10^5^ cells per ml) at a multiplicity of infection (MOI) of 10 in the humidified atmosphere with 5% CO_2_ at 37 °C for 10 min. Then, MLE-12 cells were cocultured with PBS or Reg4 (10 μg/ml) for 1 h. Nonadherent bacteria was removed by washing three times with PBS. The numbers of adherent bacteria were determined by lysing the cells with 0.1% Triton X-100 and plating serial dilutions on LB agar plates and lysed using 1% Triton X-100. For the invasion assay, Caco-2 cells were incubated with 100 ug/ml gentamycin for 1 h to kill the external bacteria and the bacterial CFU was determined as described above.

**Quantitative real-time polymerase chain reaction (qRT-PCR)**

Lung tissue was homogenized and total RNA was obtained with the RNeasy Mini kit according to the manufacturer’s instructions. The level of the genes was detected using the High-Capacity cDNA Reverse Transcription Kit and the SYBR-Green Universal Master Mix kit following the manufacturer’s recommendations. The list of the real-time PCR primers is provided in Supplemental Table 2. The relative mRNA expression levels of each gene were compared with that of Actb.

**Motility analysis of** PAO1

Motility was detected by inoculating stationary-phase PAO1 (OD_600_ = 0.6) onto the center of the semisolid LB agar (0.3%) plates following the previously described protocol (7). PAO1 were incubated with PBS or Reg4 for 1 h. Then, the mixture was applied to the center of the semisolid LB agar (0.3%) plates, and the diameters of the colonies were measured as the indication of bacterial migration.

**Flow cytometry**

MH-S cells treated with Reg4 (10μg/ml) or LPS (0.1μg/ml) or IL-4 (0.1μg/ml) for 24 h. Cells were harvested and stained with the manufacturer’s suggested concentrations of anti-CD206-APC, anti-CD80-APC antibody. After staining, the cells were assayed by the FACS Canto II flow cytometer (BD Biosciences, San Jose, CA, USA) and the data were analyzed using the FlowJo software version 8 (FlowJo, Ashland, OR, United States).

**Oxygen consumption rates measurement (OCR).**

A total of 20,000 MH-S cells were planted in an XF96 cell culture microplate. The growth media was changed to bicarbonate-free assay media with PBS or Reg4 protein (10 μg/ml) and incubated at 37 °C for 1 h in a CO_2_-free incubator. MH-S cells were run on an XF96e Analyzer for a Mito Stress Test in XF DMEM medium (pH 7.4) with 25 mM glucose, 2 mM pyruvate and 2 mM glutamine using the manufacturer’s protocol and following the standard drug concentrations of 1 µM oligomycin, 1.2 µM FCCP, 0.5 µM rotenone and 0.5 µM antimycin A.

**Extracellular acidification rates measurement (ECAR).**

Twenty thousand MH-S cells were planted in an XF96 cell culture microplate. The following day, growth media was changed to bicarbonate-free assay media with PBS or Reg4 protein (10 μg/ml) and incubated at 37 °C for 1 h in a CO_2_-free incubator. Extracellular acidification rate (ECAR) was measured using an XF96 Extracellular Flux Analyzer under basal conditions and following addition of 10 mM glucose, 1 μM Oligomycin, and 50 mM glucose analog, 2-deoxyglucose, 2DG, according to the manufacturer’s protocol.

**Immunochemistry (IHC) staining**

Immunochemistry (IHC) staining was performed as we described previously (11, 12). Briefly, left lung sections (4 μm) thick were mounted on positively-charged slides and were incubated with xylol and descending concentrations of ethanol.. After antigen retrieval, blocking was performed using 5% bovine serum albumin (BSA) for 30 min. The antibodies iNOS (#GB11119, 1:400, Servicebio, Wuhan, China) and CD206 (#GB11062, 1:500, Servicebio, Wuhan, China) were applied at their optimal concentration overnight in a wet chamber for overnight at 4 °C. The slides were rinsed in phosphate-buffered saline (PBS) and incubated with the appropriate secondary antibody for 1 hour. The slides were rinsed in PBS and hematoxylin. Morphometry were counted in 10 randomly chosen microscopic high-power fields (hpf) per sample. Each group, n = 5 - 6. The images analysis was used software Image Pro Plus (Media Cybernetics, Rockville, MD, USA).

**Supplemental Table 1. Key resources table.**

| **REAGENT or RESOURCE** | **SOURCE** | **IDENTIFIER** |
| --- | --- | --- |
| **Antibodies** | | |
| REG4 antibody | Abcam | Cat# ab204171, RRID:AB_2050246 |
| Penta·His Antibody, BSA-free | Qiagen | Cat# 34660, RRID:AB_2619735 |
| CD68 | Proteintech | Cat#66231-2-IG |
| APC-CD80 | eBioscience | Cat#17-0801-82 |
| APC-CD206 | Biolegend | Cat#141707 |
| **Bacterial and Virus Strains** | | |
| *Pseudomonas aeruginosa* | BIOBW | ATCC15692 |
| BL21(DE3) competent cell | Vazyme | Cat# C504-03 |
| MH-S cell | This study | N/A |
| MLE-12 cell | This study | N/A |
| **Chemicals, Peptides, and Recombinant Proteins** | | |
| pET-28-Reg4(NM_026328-6His) | Shanghai Genechem Co., Ltd | N/A |
| Tryptone | MKBio | Cat# MS6003-500G |
| Agarose | BioFroxx | Cat# 1110GR100 |
| Oxoid™ Yeast Extract Powder | Oxoid | Cat# LP0021B |
| Kanamycin | Merck | Cat# E004000-5G |
| Isopropyl β-D-thiogalactoside | Meilunbio | Cat# MB3026 |
| Ni-NTA Fast Start Kit | QIAGEN | Cat# 30600 |
| Vivaspin 20, 3 kDa MWCO PES | GE Healthcare | Cat# 28932358 |
| PD MidiTrap G-25 | GE Healthcare | Cat# 28918008 |
| PIERCE BCA PROTEIN ASSAY | Invitrogen | Cat# 23227 |
| Lavage needle | Genecome | Cat# GWZ-8-45 |
| EndoFree Mini Plasmid Kit II | TIANGEN | Cat# DP118-02 |
| RNeasy Mini Kit (250) | QIAGEN | Cat# 74106 |
| Tetramethylbenzidine substrate | Beyotime | Cat# P0209-100ml |
| Mouse IL-22 ELISA KIT | Shycbio | Cat# YCJL29108 |
| Latex beads, carboxylate-modified polystyrene, fluorescent yellow-green | Merck | Cat# L4655 |
| IL-4 | Shycbio | Shycbio |
| Mouse Reg4 ELISA KIT | X-Y Biotechnology | Cat# XY-REG4-Mu |
| **Experimental Models: Organisms/Strains** | | |
| C57BL/6 mice | Shanghai Jihui Laboratory Animal Care Co., Ltd | N/A |

**Supplemental Table 2. Primers for genes expression using real-time PCR.**

| **Genes** |  | **Sequence (5’-3’)** | **Size (bp)** | **NCBI Gene ID** |
| --- | --- | --- | --- | --- |
| Il6 | Forward | GACAAAGCCAGAGTCCTTCAGA | 76 | NM_001314054.1 |
|  | Reverse | TGTGACTCCAGCTTATCTCTTGG |  |  |
| Il10 | Forward | TGAAAACAAGAGCAAGGCCG | 136 | NM_010548.2 |
|  | Reverse | GCCACCCTGATGTCTCAGTT |  |  |
| Ifng | Forward | TCAAGTGGCATAGATGTGGAAGAA | 92 | NM_008337.4 |
|  | Reverse | TGGCTCTGCAGGATTTTCATG |  |  |
| Cxcl2 | Forward | GCTGTCCCTCAACGGAAGAA | 72 | NM_009140.2 |
|  | Reverse | CAGGTACGATCCAGGCTTCC |  |  |
| Ccl2 | Forward | TGCCCTAAGGTCTTCAGCAC | 150 | NM_011333.3 |
|  | Reverse | AAGGCATCACAGTCCGAGTC |  |  |
| Tlr4 | Forward | TGGTTGCAGAAAATGCCAGG | 169 | NM_021297.3 |
|  | Reverse | TCATCAGGGACTTTGCTGAGTT |  |  |
| Tnfaip3 | Forward | GGAACTGCCCAGTCTGTAGT | 74 | NM_009397.3 |
|  | Reverse | GGGTGTGCACGTCTTTCGG |  |  |
| Nfkbia | Forward | CCTGACCTGGTTTCGCTCTT | 73 | NM_010907.2 |
|  | Reverse | AGGGGGAGTAGCCTTGGTAG |  |  |
| Nfkbie | Forward | CCGACTCTCTGCTGCTGAAT | 111 | NM_008690.4 |
|  | Reverse | ATAGCAGTGGTTTGCCGGAG |  |  |
| Tnf | Forward | ATGGCCTCCCTCTCATCAGT | 97 | [NM_013693.3](https://www.ncbi.nlm.nih.gov/entrez/viewer.fcgi?db=nucleotide&id=518831586) |
|  | Reverse | TTTGCTACGACGTGGGCTAC |  |  |
| Il22 | Forward | TGCGATCTCTGATGGCTGTC | 90 | [NM_016971.2](https://www.ncbi.nlm.nih.gov/entrez/viewer.fcgi?db=nucleotide&id=255958194) |
|  | Reverse | ACAGGGCAATGAGAAGCAGG |  |  |
| Reg4 | Forward | AACCTGCCTGTGTGGATTGG | 135 | NM_026328.2 |
|  | Reverse | GTTCATCTCAGCGCAATGCC |  |  |
| Il1b | Forward | TCAGGCAGGCAGTATCACTCA | 75 | NM_008361.4 |
|  | Reverse | GGAAGGTCCACGGGAAAGAC |  |  |
| Actb | Forward | CACTGTCGAGTCGCGTCC | 102 | NM_007393.5 |
|  | Reverse | CGCAGCGATATCGTCATCCA |  |  |

**Supplemental Figure 1.** **Gene expression altered by Reg4.**

The mRNA expression levels of Cxcl2, Ccl2, Tlr4, Tnfaip3, Nfkbie, and Nfkbia using q-RT-PCR. Values were normalized to Actb expression.


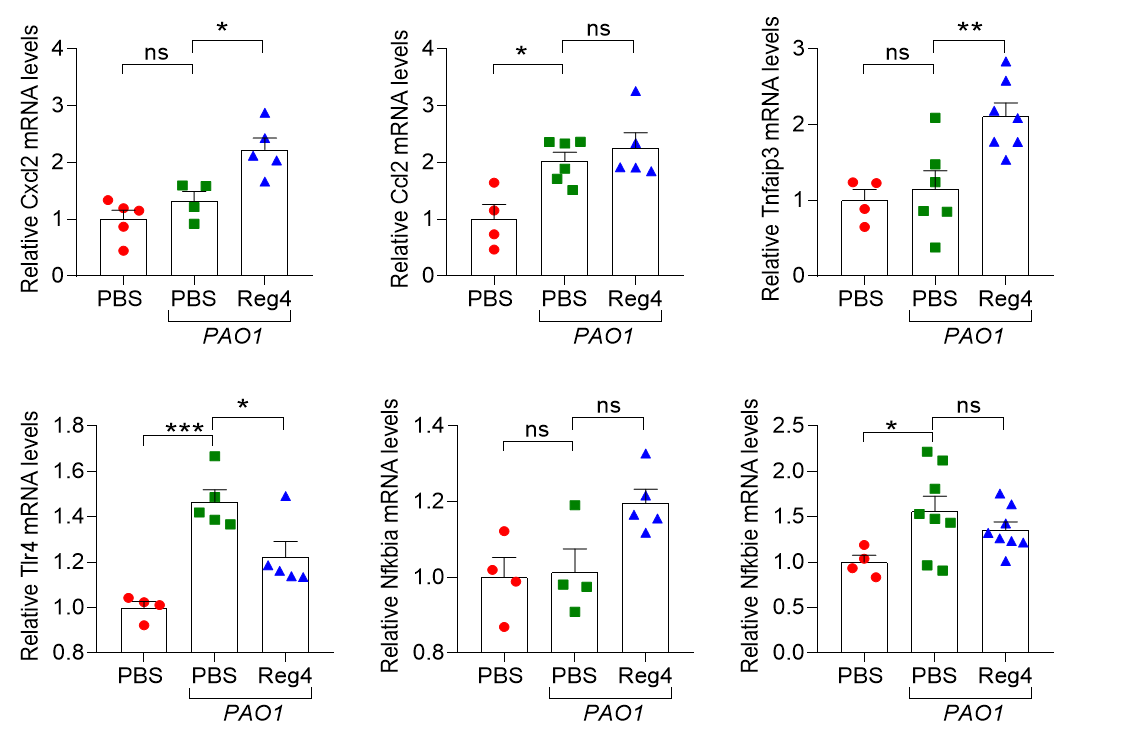
Data are mean ± SEM; one-way ANOVA and two-way ANOVA were used for statistical analysis; * *p* < 0.05; ** *p* < 0.01; *** *p* < 0.001.

**Supplemental Figure 2. Reg4 reduces adhesion and invasion of PAO1 on intestinal epithelial cells**

(A) Representative images of CFUs for the lungs, bronchoalveolar lavage fluid (BALF), and spleen from PBS-treated, PAO1-infected, and PAO1-infected and Reg4-treated mice.

(B) Representative images of CFUs for adhesion of PAO1 to murine lung epithelial (MLE-12) cells treated with PBS or Reg4.

(C) Representative images of CFUs for invasion of PAO1 in MLE-12 cells treated with PBS or Reg4.


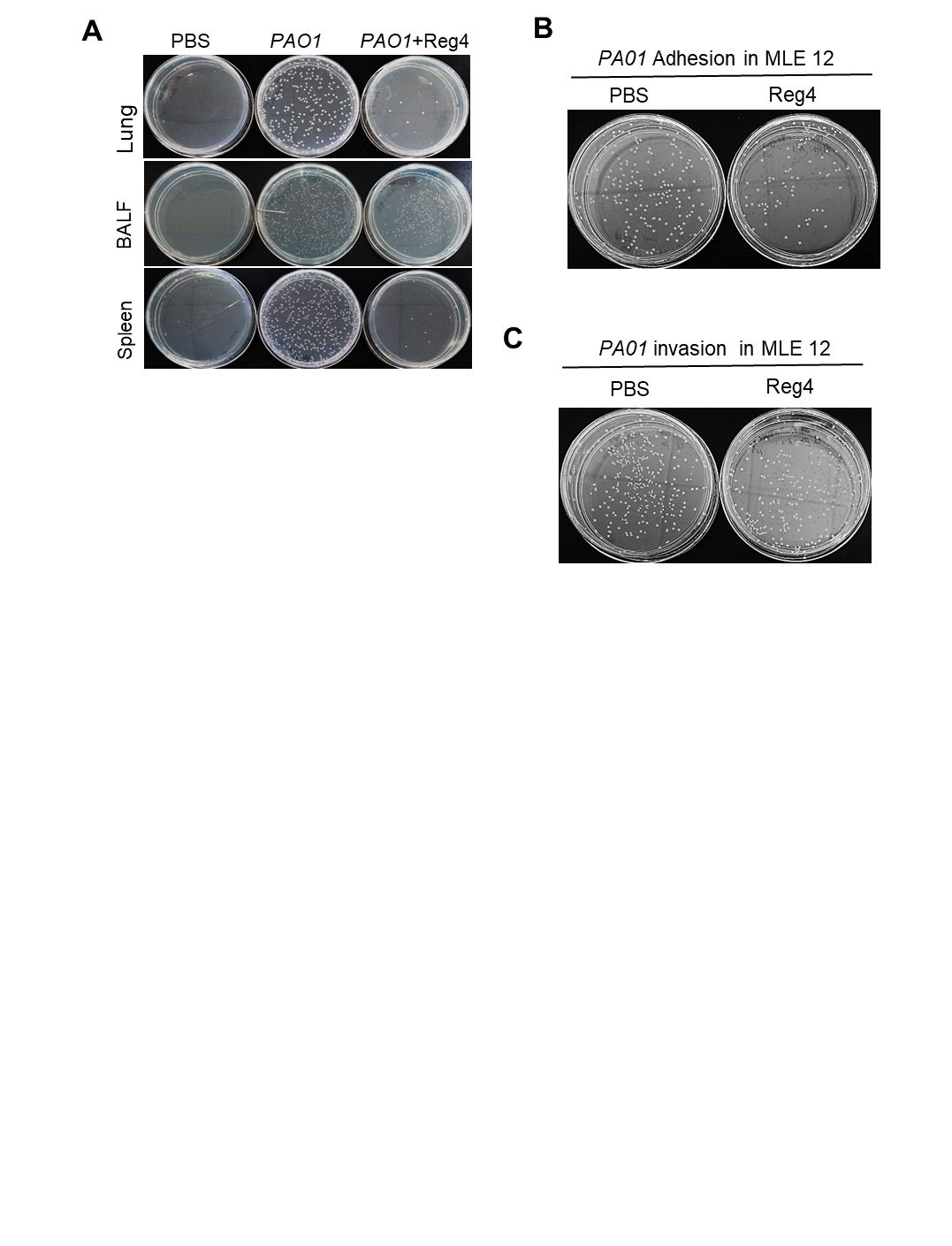


**Supplementary References**

1. Jarret A, Jackson R, Duizer C, Healy ME, Zhao J, Rone JM, Bielecki P, Sefik E, Roulis M, Rice T, Sivanathan KN, Zhou T, Solis AG, Honcharova-Biletska H, Velez K, Hartner S, Low JS, Qu R, de Zoete MR, Palm NW, Ring AM, Weber A, Moor AE, Kluger Y, Nowarski R, Flavell RA. 2020. Enteric Nervous System-Derived IL-18 Orchestrates Mucosal Barrier Immunity. Cell 180:50-63 e12.

2. Ho MR, Lou YC, Lin WC, Lyu PC, Huang WN, Chen C. 2006. Human pancreatitis-associated protein forms fibrillar aggregates with a native-like conformation. J Biol Chem 281:33566-76.

3. Gaur P, Hada V, Rath RS, Mohanty A, Singh P, Rukadikar A. 2023. Interpretation of Antimicrobial Susceptibility Testing Using European Committee on Antimicrobial Susceptibility Testing (EUCAST) and Clinical and Laboratory Standards Institute (CLSI) Breakpoints: Analysis of Agreement. Cureus 15:e36977.

4. Giske CG, Turnidge J, Canton R, Kahlmeter G, Committee ES. 2022. Update from the European Committee on Antimicrobial Susceptibility Testing (EUCAST). J Clin Microbiol 60:e0027621.

5. Humphries R, Bobenchik AM, Hindler JA, Schuetz AN. 2021. Overview of Changes to the Clinical and Laboratory Standards Institute Performance Standards for Antimicrobial Susceptibility Testing, M100, 31st Edition. J Clin Microbiol 59:e0021321.

6. Minami M, Takase H, Nakamura M, Makino T. 2019. Methanol extract of Lonicera caerulea var. emphyllocalyx fruit has

anti-motility and anti-biofilm activity against enteropathogenic

Escherichia coli. Drug Discoveries & Therapeutics 13:335-342.

7. Okumura R, Kurakawa T, Nakano T, Kayama H, Kinoshita M, Motooka D, Gotoh K, Kimura T, Kamiyama N, Kusu T, Ueda Y, Wu H, Iijima H, Barman S, Osawa H, Matsuno H, Nishimura J, Ohba Y, Nakamura S, Iida T, Yamamoto M, Umemoto E, Sano K, Takeda K. 2016. Lypd8 promotes the segregation of flagellated microbiota and colonic epithelia. Nature 532:117-21.

8. Malleret B, El Sahili A, Tay MZ, Carissimo G, Ong ASM, Novera W, Lin J, Suwanarusk R, Kosaisavee V, Chu TTT, Sinha A, Howland SW, Fan Y, Gruszczyk J, Tham W-H, Colin Y, Maurer-Stroh S, Snounou G, Ng LFP, Chan JKY, Chacko A-M, Lescar J, Chandramohanadas R, Nosten F, Russell B, Rénia L. 2021. Plasmodium vivax binds host CD98hc (SLC3A2) to enter immature red blood cells. Nature Microbiology 6:991-999.

9. Files DC, Liu C, Pereyra A, Wang ZM, Aggarwal NR, D'Alessio FR, Garibaldi BT, Mock JR, Singer BD, Feng X, Yammani RR, Zhang T, Lee AL, Philpott S, Lussier S, Purcell L, Chou J, Seeds M, King LS, Morris PE, Delbono O. 2015. Therapeutic exercise attenuates neutrophilic lung injury and skeletal muscle wasting. Sci Transl Med 7:278ra32.

10. Horstmann JA, Lunelli M, Cazzola H, Heidemann J, Kuhne C, Steffen P, Szefs S, Rossi C, Lokareddy RK, Wang C, Lemaire L, Hughes KT, Uetrecht C, Schluter H, Grassl GA, Stradal TEB, Rossez Y, Kolbe M, Erhardt M. 2020. Methylation of Salmonella Typhimurium flagella promotes bacterial adhesion and host cell invasion. Nat Commun 11:2013.

11. Xiao Y, Yan W, Lu Y, Zhou K, Cai W. 2018. Neurotensin contributes to pediatric intestinal failure-associated liver disease via regulating intestinal bile acids uptake. EBioMedicine 35:133-141.

12. Tian X, Wang Y, Lu Y, Wu B, Chen S, Du J, Cai W, Xiao Y. 2022. Metabolic regulation of cholestatic liver injury by D-2-hydroxyglutarate with the modulation of hepatic microenvironment and the mammalian target of rapamycin signaling. Cell Death Dis 13:1001.
